# The actin-bundling protein, PLS3, is part of the mechanoresponsive machinery that regulates osteoblast mineralization

**DOI:** 10.3389/fcell.2023.1141738

**Published:** 2023-11-27

**Authors:** Samantha M. Chin, Carmela Unnold-Cofre, Teri Naismith, Silvia Jansen

**Affiliations:** Department of Cell Biology and Physiology, Washington University in St. Louis, Saint Louis, MO, United States

**Keywords:** osteoblast mineralization, PLS3, childhood osteoporosis, mechanosensation, substrate stiffness

## Abstract

Plastin-3 (PLS3) is a calcium-sensitive actin-bundling protein that has recently been linked to the development of childhood-onset osteoporosis. Clinical data suggest that PLS3 mutations lead to a defect in osteoblast function, however the underlying mechanism remains elusive. To investigate the role of PLS3 in bone mineralization, we generated MC3T3-E1 preosteoblast cells that are stably depleted of PLS3. Analysis of osteogenic differentiation of control and PLS3 knockdown (PLS3 KD) cells showed that depletion of PLS3 does not alter the first stage of osteoblast mineralization in which a collagen matrix is deposited, but severely affects the subsequent mineralization of that matrix. During this phase, osteoblasts heavily rely on mechanosensitive signaling pathways to sustain mineral deposition in response to increasing stiffness of the extracellular matrix (ECM). PLS3 prominently localizes to focal adhesions (FAs), which are intricately linked to mechanosensation. In line with this, we observed that depletion of PLS3 rendered osteoblasts unresponsive to changes in ECM stiffness and showed the same cell size, FA lengths and number of FAs when plated on soft (6 kPa) *versus* stiff (100 kPa) substrates in contrast to control cells, which showed an increased in each of these parameters when plated on 100 kPa substrates. Defective cell spreading of PLS3 KD cells on stiff substrates could be rescued by expression of wildtype PLS3, but not by expression of three PLS3 mutations that were identified in patients with early onset osteoporosis and that have aberrant actin-bundling activity. Altogether, our results show that actin-bundling by PLS3 is part of the mechanosensitive mechanism that promotes osteoblast mineralization and thus begins to elucidate how PLS3 contributes to the development of bone defects such as osteoporosis.

## Introduction

Osteoporosis is the most common bone disorder in the world and represents a significant clinical and societal burden due to related bone fractures (Blume and Curtis, 2011; Coughlan and Dockery, 2014). Although classically viewed as an age-related disorder, osteoporosis now more commonly describes a general condition of low bone mineral density that can arise in children as well as adults (Ward et al., 2016; Weaver et al., 2016). In either case, the characteristic brittle bone phenotype of osteoporosis is the result of imbalances between osteoblasts that deposit bone, osteoclasts that remove bone, and osteocytes that translate mechanical load into chemical signals that regulate osteoblast and osteoclast function (Florencio-Silva et al., 2015). To date, osteoporosis is mainly treated by curbing bone resorption by osteoclasts, however the severe side effects and concerns about the long-term efficacy of these compounds call for novel targets to treat osteoporosis (Khosla and Hofbauer, 2017; Lloyd et al., 2017). Moreover, healthy bone maintenance requires new bone formation (Buckwalter and Cooper, 1987), and thus there is increasing interest in proteins that can stimulate osteoblast function.

The calcium-sensitive actin-bundling protein, Plastin-3 (PLS3, also called Fimbrin), has emerged as an interesting new target to regulate bone formation and bone maintenance (van Dijk et al., 2013). This is supported by its predominant expression in osteoblasts and osteocytes (Lin et al., 1993; Delanote et al., 2005; Fahiminiya et al., 2014; Wesseling-Perry et al., 2017; Yorgan et al., 2020), as well as by the identification of over 20 pathogenic PLS3 variants in children as well as adults with early onset X-linked osteoporosis (van Dijk et al., 2013; Fahiminiya et al., 2014; Laine et al., 2015; Kämpe et al., 2017a; Kämpe et al., 2017b; Kannu et al., 2017; Balasubramanian et al., 2018; Costantini et al., 2018; Wang et al., 2018; 2020; Shao et al., 2019). In line with this, transient depletion of PLS3 in zebrafish and whole genome PLS3 knockout mice show defects in skeletal development and reduced bone mass, however the underlying mechanisms have not been characterized, including whether this requires the actin-regulatory function of PLS3 (van Dijk et al., 2013; Fahiminiya et al., 2014; Neugebauer et al., 2018; Yorgan et al., 2020). These studies did suggest that the role of PLS3 in bone health is related to a role in osteoblast mineralization (Fahiminiya et al., 2014; Yorgan et al., 2020). Osteoblasts are mesenchymal lineage cells that undergo osteogenic differentiation to deposit bone (Dirckx et al., 2013; Capulli et al., 2014; Rutkovskiy et al., 2016; Blair et al., 2017). This process is characterized by two main phases: 1) production of an organic matrix primarily consisting of collagen I, and 2) subsequent mineralization of the collagen matrix by hydroxyapatite crystals formed in matrix vesicles (MVs) (Dirckx et al., 2013; Blair et al., 2017). The latter are membrane-bound vesicles secreted by mature osteoblasts that are enriched in enzymes (TNAP, ENPP1, etc.), calcium channels, and phosphate transporters, which promote the accumulation of high intravesicular concentrations of calcium and phosphate that act as a nidus for the formation of hydroxyapatite crystals. These crystals assemble within the MV and eventually grow through the MV membrane to form mineralized nodules embedded within the collagen matrix (Dirckx et al., 2013).

Differentiation and mineral deposition by pre-osteoblast cells are driven by a number of factors including the organization of the extracellular matrix (ECM), bioactive molecules (growth factors, ions, vitamins), and mechanical stimuli (Rutkovskiy et al., 2016; Blair et al., 2017). Previous studies have demonstrated that pre-osteoblast cells are highly sensitive to mechanical cues, where an increase in ECM stiffness due to gradual mineralization of the collagen scaffold contributes to a feedforward mechanism that fuels further mineral deposition by osteoblasts (Keogh et al., 2010; Mullen et al., 2014; 2015; Zhang et al., 2018). Although the molecular details of this process are not entirely understood, both focal adhesions (FAs) and actin stress fibers have been shown to play an important role (Mullen et al., 2014). In line with this, knockdown mice models of several proteins that have a structural and/or signaling function in FAs are defective in osteoblast differentiation and bone mineralization (Xiao et al., 2000; Leucht et al., 2007; Dejaeger et al., 2017; Wang et al., 2019; Zhao et al., 2021). A direct link between the actin cytoskeleton and mechanosensation has been unequivocally established in many different cell types, including osteoblasts and osteocytes (Fletcher and Mullins, 2010; Chang and Knothe Tate, 2011; Kuo et al., 2015; Ohashi et al., 2017; Uda et al., 2017; Martino et al., 2018; Gould et al., 2021; Esposito et al., 2022). In case of bone cells, the role of the actin cytoskeleton has been mostly studied in response to fluid shear stress and not in response to mineralization of the ECM (Kuo et al., 2015; Uda et al., 2017; Garbett et al., 2020). However, the actin cytoskeleton has been shown to be an integral part of the mechanosensitive machinery that drives osteogenic stem cell differentiation in response to increasing stiffness (Keogh et al., 2010; Mullen et al., 2015; Olivares-Navarrete et al., 2017; Zhang et al., 2018). In line with this, osteoblast mineralization is disrupted in the presence of agents that stabilize or induce disassembly of actin filaments (Zouani et al., 2013; Chen et al., 2015; Suzuki et al., 2019). This emphasizes that actin filament dynamics are required for osteoblast mineralization, raising the question how actin-binding proteins, such as PLS3, contribute to osteoblast differentiation and function.

PLS3 (also known as T-plastin) is one of three isoforms of the PLS3/fimbrin family expressed in vertebrates. PLS3 is the most ubiquitously expressed isoform and is found in all solid tissues, whereas Plastin-1 is only found in specialized structures such as the stereocilia in the inner ear epithelium and the microvilli of the small intestine and kidney epithelium, and Plastin-2 is mainly expressed in hematopoietic cells (Lin et al., 1993). All plastins share a common domain structure consisting of two EF-hand domains followed by two actin-binding domains (ABDs) that are each comprised of a pair of calponin-homology domains. The latter bind strongly to actin filaments and their tandem arrangement enables F-actin bundling and crosslinking. This activity is regulated by binding of calcium to the N-terminal EF-hands, which inhibits actin-bundling by PLS3, but not actin-binding (Schwebach et al., 2017; Khassan et al., 2019). PLS3 has been shown to play a role in fundamental cellular processes, including cell migration, cell-cell contact and endocytosis, which further explain how PLS3 contributes to cancer metastasis, cardiovascular defects, and spinal muscular atrophy (Hagiwara et al., 2011; Brun et al., 2014; Wottawa et al., 2017; Garbett et al., 2020; Pan et al., 2020). It remains unclear whether these PLS3 functions contribute to osteoblast physiology and bone maintenance, although a recent biochemical analysis of three osteopathogenic PLS3 mutants did show that the underlying mechanism at least requires the actin-bundling activity of PLS3 (Schwebach et al., 2020).

In this study, we aim to gain insight into how PLS3 contributes to the development of childhood osteoporosis by investigating the role of calcium-sensitive actin bundling of PLS3 in osteoblast mineralization. Using MC3T3-E1 cells, which mimic osteoblast differentiation and mineralization, we show that depletion of PLS3 specifically leads to a defect in mineralization of the deposited collagen matrix. Immunohistochemical analysis next showed prominent localization of endogenous PLS3 at the transition of focal adhesions (FAs) and actin stress fibers, suggesting that PLS3 might be part of the mechanosensitive machinery that drives osteoblast maturation and mineralization. In line with this, PLS3 depletion did not affect cell adhesion and spreading in cells plated on glass, which mimics fully mineralized bone; but severely disrupted spreading of cells on substrates with a stiffness corresponding to mineralizing bone. This phenotype could be rescued by overexpression of WT PLS3 but not by overexpression of three osteopathogenic PLS3 mutants. Altogether, our data suggest that PLS3 contributes to osteoblast mineralization by regulating the mechanosensitive response of osteoblasts to increasing stiffness of their ECM, which could explain the correlation between PLS3 mutation and early onset osteoporosis.

## Results

### Loss of PLS3 impairs mineralization but not deposition of the collagen matrix

**FIGURE 1:**
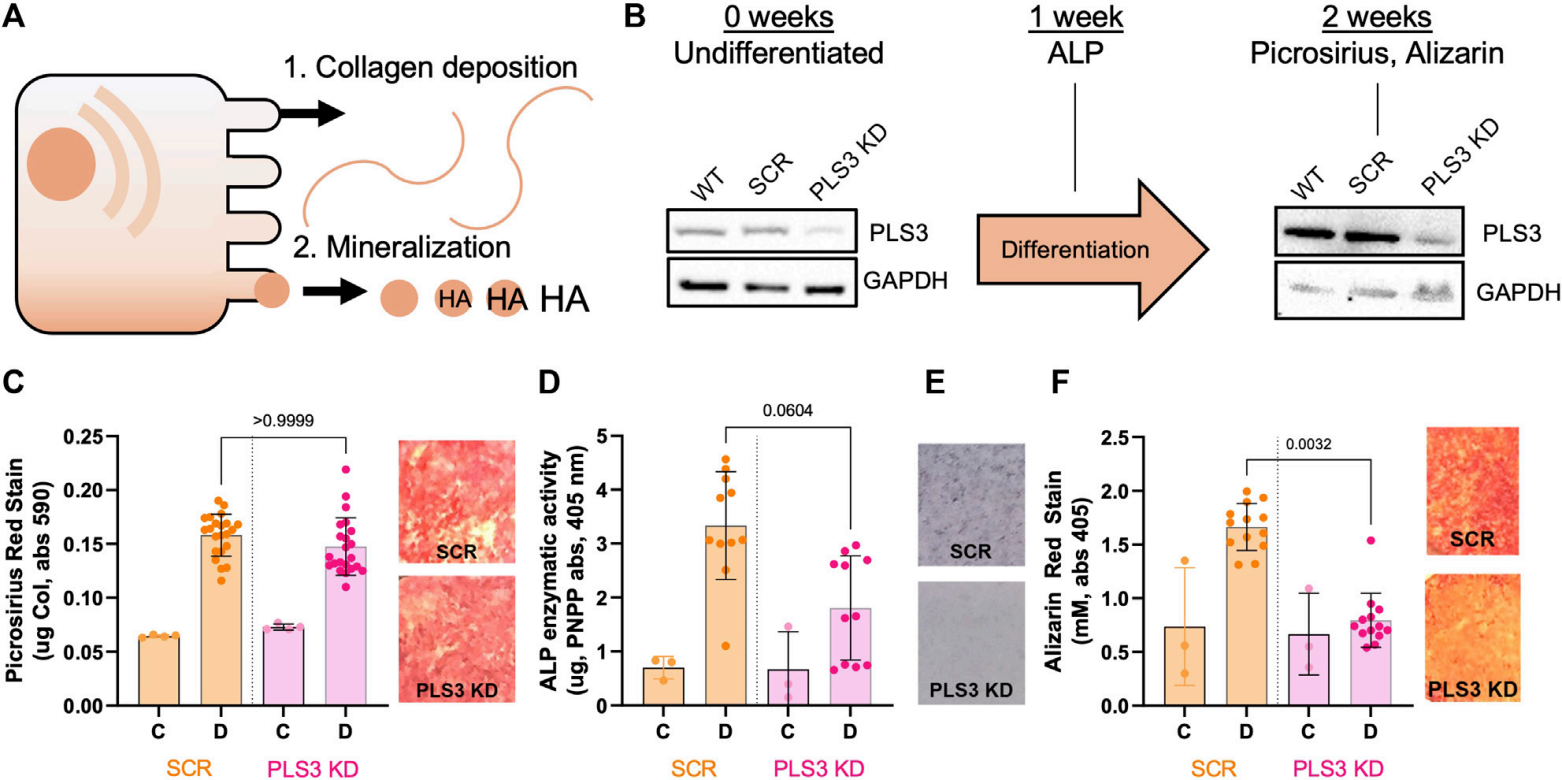
Loss of PLS3 impairs osteoblast mineralization but not deposition of the collagen matrix**. (A)** Model demonstrating the two phases of osteoblast differentiation**. (B)** Schematic overview showing differentiation timeline and endpoints for different osteogenic assays shown in C-F. Western blots show PLS3 expression in WT, Scrambled (Scr) and PLS3 knockdown (PLS3 KD) MC3T3 cells before and after 2 weeks of osteogenic differentiation. GAPDH was included as a loading control**. (C)** Distribution of spectrophotometric quantification of fibrillar collagen deposited by osteoblasts under control **(C)** and differentiation **(D)** conditions for SCR and PLS3 KD cells and visualized with picrosirius red staining (left). Data was analyzed from three independent experiments with ≥5 wells of differentiating condition per experiment. Representative images of picrosirius red staining of differentiated Scr and PLS3 KD cells are shown next to the graph**. (D)** Distributions of spectrophotometric quantification of alkaline phosphatase activity based on conversion of PNPP substrate under control **(C)** and differentiation **(D)** conditions for SCR and PLS3 PLS3 KD cells after 1 week of differentiation. Data was analyzed from three independent experiments with ≥2 wells of differentiating condition per experiment**. (E)** Representative images of alkaline phosphatase activity using NBT/BCIP stain of differentiated SCR and PLS3 KD cells after 2 weeks of differentiation**. (F)** Distributions (left) and representative images (right) of spectrophotometric quantification of matrix calcification visualized with alizarin red stain under control **(C)** and differentiation **(D)** conditions for SCR and PLS3 KD cells after 2 weeks of differentiation. Data was analyzed from three independent experiments with ≥3 wells of differentiating condition per experiment. Statistical significance was determined using one-way ANOVA and Kruskal Wallis multiple comparison tests.

To investigate the role of PLS3 in osteoblast differentiation and mineralization, we generated MC3T3-E1 pre-osteoblast cells that are stably depleted of PLS3 using a previously published lentiviral short-hairpin approach (Dor-On et al., 2017). We specifically chose MC3T3 cells because this cell type has been shown to undergo osteogenic differentiation and mineral deposition under osteogenic conditions, mimicking primary osteoblast mineralization (Addison et al., 2015) (Figure 1A). Western blot analysis confirmed depletion of PLS3 in knockdown cells (PLS3 KD) in comparison to both wild type MC3T3 cells (WT) as well as MC3T3 cells that are stably expressing a non-targeting shRNA (Scrambled or Scr, Figure 1B). Next, we analyzed the ability of PLS3 KD cells to deposit a mineralized matrix. For this purpose, cells were grown in the presence of ascorbic acid (100 μM) and β-glycerophosphate (2 mM) for 7–14 days with media changes every 3 days. Osteogenic differentiation and mineralization were examined by three standard assays, including histological staining for collagen using picrosirius red, alkaline phosphatase (ALP) using NBT/BCIP staining, and hydroxyapatite (HA) mineral deposition using Alizarin red (Figures 1C,E,F). Independently, ALP activity was measured in cell lysates derived from differentiated and undifferentiated cells (Figure 1E). As expected, these assays showed that collagen secretion, ALP activity and HA deposition were all increased in differentiated *versus* undifferentiated Scr cells (Figures 1C–F). It should be noted that we observed similar changes in these parameters between differentiated and undifferentiated WT cells, indicating that lentiviral treatment and antibiotic selection did not affect the differentiation and mineralization of our stable cell lines (Supplementary Figures S1A-D).

As PLS3 protein levels have been shown to increase about 2-fold during MC3T3 differentiation (Fahiminiya et al., 2014), we first confirmed by Western blot that PLS3 was depleted to the same extent under differentiated and undifferentiated conditions (Figure 1B). Next, we examined the osteogenic differentiation and mineralization of PLS3 KD cells compared to Scr cells. Our results demonstrate that collagen secretion of PLS3 KD cells was similar to that of Scr cells (Figure 1C). Interestingly, ALP activity was slightly, but not significantly, decreased in PLS3 KD cells, suggesting that downstream mineral deposition might be affected (Figures 1D, E). In line with this, HA mineral deposition was strongly impaired in PLS3 KD cells after 3 weeks (Figure 1F). Analysis of the hydroxyapatite deposition at earlier timepoints showed that PLS3 KD cells produce mineral during the first week of differentiation, albeit less than WT or Scr cells (Supplementary Figure S1E). However, whereas WT and Scr cells ramp up mineral production during the second and third week of differentiation, HA deposition was severely hampered in PLS3 KD cells (Supplementary Figure S1E). This suggests that PLS3 KD cells initially respond to osteogenic differentiation and produce an organic collagen matrix that serves as the scaffold for mineral deposition, however, subsequently fail to properly mineralize the deposited matrix into osteoid.

### PLS3 KD modestly affects osteoblast adhesion to glass

**FIGURE 2:**
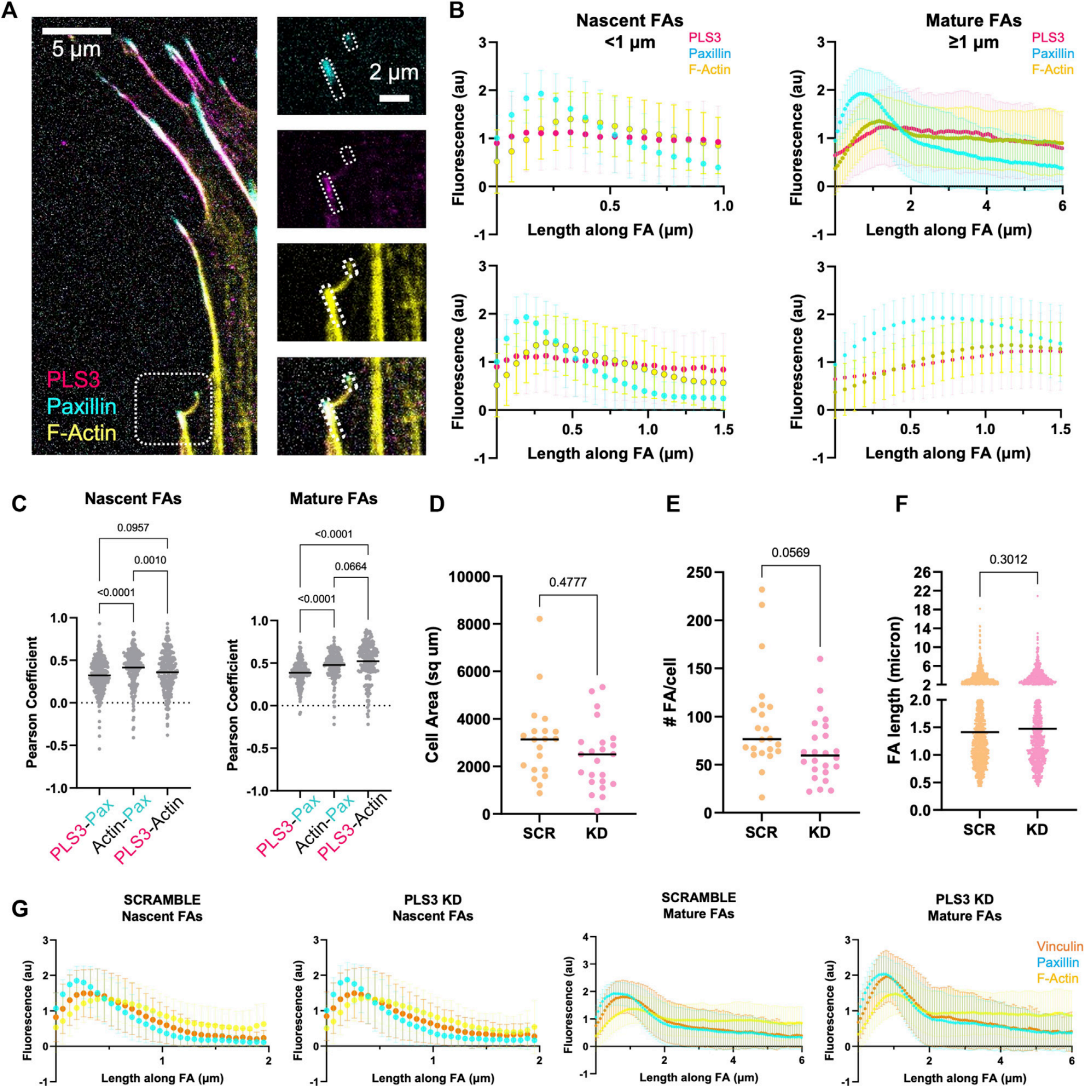
PLS3 KD modestly affects osteoblast adhesion to glass**. (A)** Representative confocal images of MC3T3-E1 pre-osteoblast cells immunostained for PLS3 (magenta), paxillin (cyan), and phalloidin to visualize F-actin (yellow). The small and larger boxed area in the insets on right show an example nascent and mature FA, respectively **(B)** Line scans along the length of either nascent (<1 micron, left) or mature (≥1 micron, right) FAs as depicted in A, showing PLS3 intensity (magenta), paxillin intensity (cyan), and phalloidin-stained F-actin intensity (yellow). Second row of line scans compares the first 1.5 micron of the analyzed FAs in nascent and mature FAs. Each line scan is the average of 1,459 nascent FAs or 588 mature FAs. FAs were analyzed from three independent experiments with ≥5 cells per experiment. **(C)** Distribution of the Pearson’s correlation coefficient of individual FAs showing colocalization between PLS3 and paxilin, paxillin and actin and PLS3 and actin. 1,459 nascent FAs or 588 mature FAs were analyzed. FAs were collected from three independent experiments with ≥5 cells per experiment. Statistical significance was determined using one-way ANOVA on ranks and Kruskal Wallis multiple comparison tests. **(D–F)** Distributions of cell area **(D)**, number of FAs **(E)** and individual FA length **(F)** in SCR and PLS3 KD cells. Cells were analyzed from three independent experiments with ≥5 cells per experiment. FAs were collected from three independent experiments with ≥5 cells per experiment. 1,697 FAs were analyzed for SCR cells and 1,383 FAs were analyzed for PLS3 KD cells. Statistical significance was determined using one-way ANOVA on ranks and Kruskal Wallis multiple comparison tests **(G)** Line scans along the length of either nascent (<1 micron) or mature (≥1 micron) FAs showing vinculin intensity (orange), paxillin intensity (cyan), and phalloidin-stained F-actin intensity (yellow) based on confocal images of immunostained SCR or PLS3 KD cells. Each line scan is the average of 1,225 nascent and 805 mature FAs for SCR and 904 nascent and 553 mature FAs for PLS3 KD cells from three independent experiments with ≥5 cells per experiment.

The process of osteoblast mineralization is not well understood, but it is known to be mechanosensitive, where the increase in extracellular matrix (ECM) stiffness due to gradual mineralization of the collagen lattice functions as a feedforward mechanism that stimulates HA deposition until fully mineralized bone is formed (Keogh et al., 2010; Mullen et al., 2015; Olivares-Navarrete et al., 2017; Zhang et al., 2018). To sense such changes in ECM rigidity and translate them into an appropriate intracellular signal, cells make use of focal adhesions (FAs) and actin stress fibers. Interestingly, immunofluorescent staining of MC3T3 cells showed that endogenous PLS3 prominently localizes to FAs (Figure 2A). Line scans along the length of FAs co-stained for paxillin to mark FAs and for phalloidin to visualize actin, further demonstrated that PLS3 specifically accumulates at the proximal end of mature FAs (>1 micron), where they transition into actin stress fibers (Figure 2B). By contrast, the PLS3 signal was more diffuse in nascent adhesions (<1 micron) (Figure 2B left). In agreement with the line scans, Pearson’s correlation analysis of FAs showed stronger overlap of PLS3 with actin than paxillin in mature FAs (Figure 2C). These observations indicate that PLS3 contributes to the organization and formation of the initial region of the stress fiber and thus might play a role in FA maturation and turnover, and by extension sensing of the ECM. To examine this, we performed immunofluorescence staining of endogenous paxillin and actin in Scr and PLS3 KD cells plated on plain glass or glass coated with diluted Matrigel. Next, we analyzed cell size and several FA parameters. PLS3 KD cells were on average smaller in size than Scr cells, although the difference was too small to be statistically significant (Figure 2D and Supplementary Figure S2A). In line with this, the number of FAs per cells was slightly, but not significantly, lower in PLS3 KD cells and no difference was observed in the distribution of FA lengths between PLS3 KD and Scr cells (Figures 2E,F and S2B-C). In order to gain more insight into the organization of the FAs, we also performed immunofluorescent staining of endogenous paxillin and vinculin, a FA protein that connects the FA to the actin cytoskeleton (Goldmann et al., 1996; Bays and DeMali, 2017). This analysis demonstrated no difference in vinculin recruitment to FAs or changes in the spatial organization of vinculin relative to paxillin (Figure 2G). Altogether, this suggests that the effects of PLS3 on FA assembly and turnover are minimal when plated on glass, which is equivalent to fully mineralized bone. Similar observations were made for Scr and PLS3 KD cells plated on glass coated with diluted Matrigel, further showing that these cells behave the same, even in the presence of ECM. However, during mineralization, differentiating osteoblasts interact with an ECM that dramatically changes in stiffness, raising the question whether PLS3 KD cells might respond differently when plated on substrates that more faithfully mimic the stiffness range of unmineralized and/or mineralizing bone.

### PLS3 is involved in sensing substrate stiffness

**FIGURE 3:**
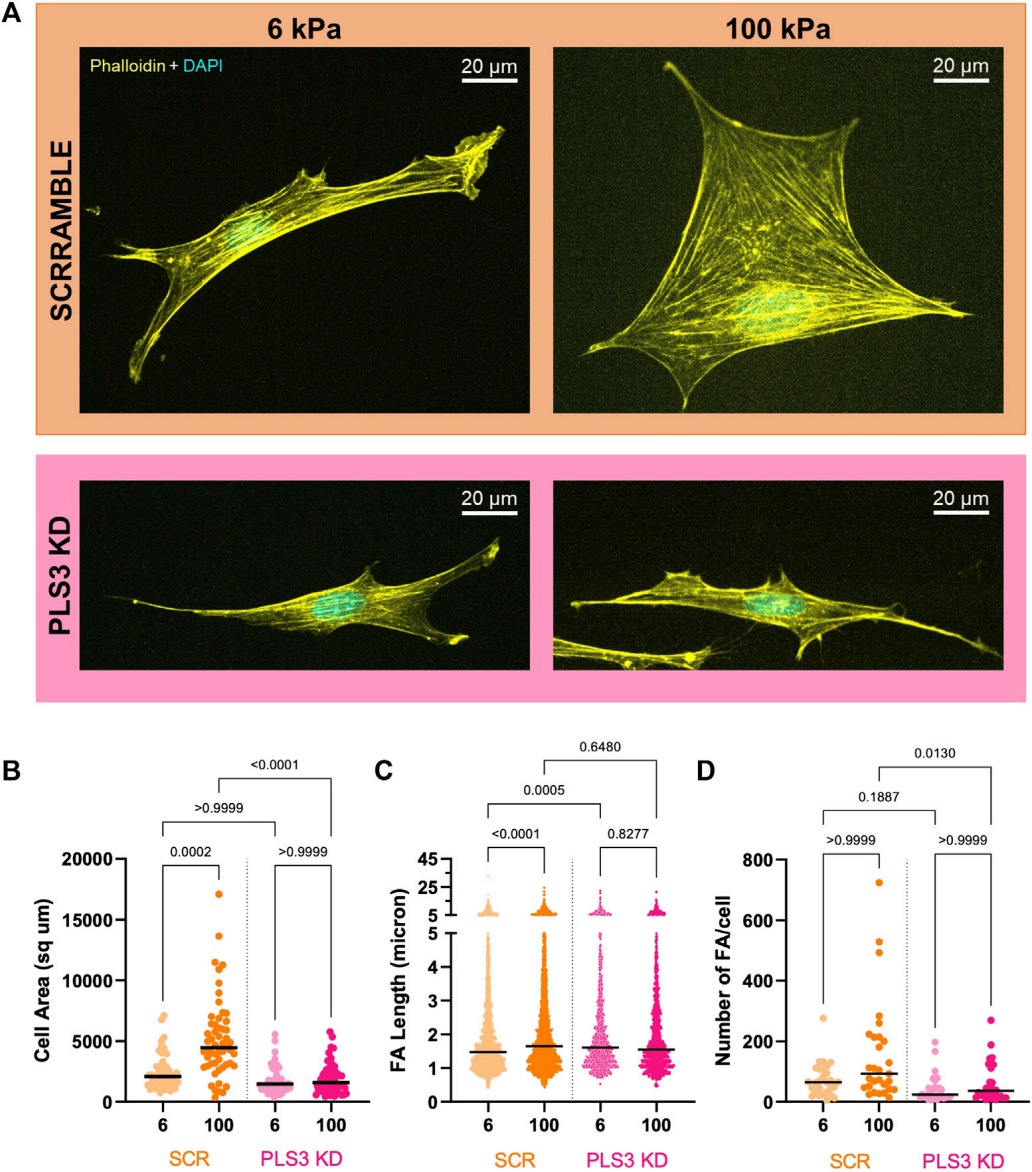
PLS3 is involved in sensing substrate stiffness**. (A)** Representative confocal images of SCR or PLS3 KD cells plated on polyacrylamide hydrogels of the indicated stiffness. Cells were immunostained with DAPI (cyan) and phalloidin (yellow). **(B–D)** Distributions of cell area **(B)**, individual FA length **(C)** and number of FAs **(D)** of SCR and PLS3 KD cells plated on polyacrylamide hydrogels of the indicated stiffness. Cells were analyzed from three independent experiments with ≥10 cells per experiment for cell size, with ≥9 cells per experiment and at least 1,093 focal adhesions per condition for FA length and with ≥8 cells per experiment for Number of FAs. Statistical significance was determined using one-way ANOVA and Kruskal Wallis multiple comparison tests.

To test whether PLS3 plays a role in sensing substrate stiffness in osteoblasts, we next analyzed cell adhesion and spreading on substrates with different stiffnesses. For this purpose, Scr and PLS3 KD cells were plated on Matrigel-coated polyacrylamide hydrogels that were shown to have stiffnesses of 6 kPa and 100 kPa and that recapitulate the physiological stiffness of endothelial tissue or unmineralized bone (aka osteoid), respectively (Engler et al., 2006; Tse and Engler, 2010). As reported previously, we observed that the size of Scr control cells correlated with substrate stiffness, where cells seeded on stiffer (100 kPa) substrates were larger than those on softer substrates (6 kPa) (Figures 3A, B) (Engler et al., 2006; Mullen et al., 2015). Immunostaining for paxillin further showed that the number and length of FAs in control cells also increased with increasing stiffness (Figures 3C, D). By contrast, PLS3 KD cells were unresponsive to changes in ECM stiffness and showed similar cell sizes when plated on 6 kPa *versus* 100 kPa (Figure 3B). In addition, analysis of FA size and number showed that these parameters did not change between PLS3 KD cells plated on soft or stiff substrates (Figures 3C, D). In comparison to Scr control cells, PLS3 KD cells showed the same number of slightly longer FAs when plated on 6 kPa, however this did not affect overall cell size (Figures 3B–D). More pronounced differences were observed between Scr and PLS3 KD cells plated on 100 kPa, with PLS3 KD cells being significantly smaller in size and displaying fewer FAs (Figures 3B–D). This effect was not limited to substrates with a stiffness corresponding to mineralizing bone, but was also observed for PLS3 KD cells plated on substrates with intermediate stiffnesses (10 and 35 kPa) (Supplementary Figure S3). Overall, this demonstrates that depletion of PLS3 affects how MC3T3 cells interact with different ECM stiffnesses. Our data further shows that PLS3 KD cells particularly fail to respond properly to substrates that mimic unmineralized bone, which suggests that PLS3 is part of the mechanosensitive machinery that regulates osteoblast mineralization in response to increasing stiffness of the collagen matrix.

### Osteogenic PLS3 mutants fail to rescue cell spreading on stiff substrates

**FIGURE 4:**
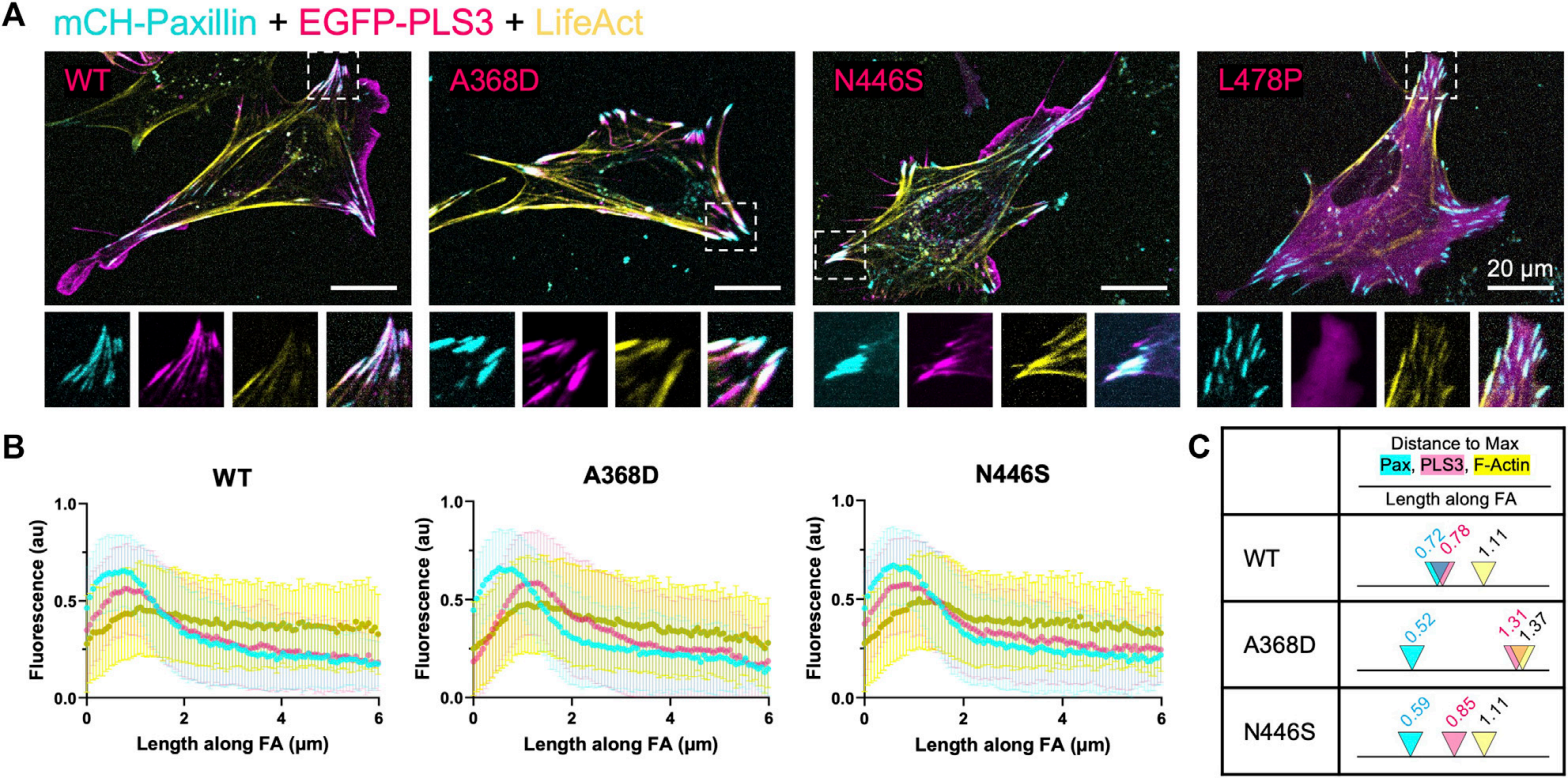
Osteopathogenic PLS3 mutants display distinct localizations in FAs **(A)** Representative live cell confocal images of PLS3 KD cells expressing mCherry-paxillin (cyan), wildtype EGFP-PLS3 or indicated EGFP-tagged osteopathogenic PLS3 mutant (magenta), and BFP-lifeact (yellow). **(B)** Line scans along the length of mature FAs showing the intensity of mCherry-paxillin (cyan), wildtype or osteopathogenic mutant of EGFP-PLS3 (magenta), and BFP-lifeact (yellow). Each line scan is the average of 595 FAs for wildtype PLS3, 489 FAs for PLS3 A468D, and 514 FAs for PLS3 N446S. FAs were analyzed from three independent experiments with ≥5 cells per experiment. **(C)** Model comparing the distance to the max peak along mature FAs for WT PLS3, PLS3 A368D, and PLS3 N446S to scale with arrowheads indicating the max peak and the distance along the FA marked above.

To examine whether the defective response of PLS3 KD cells to substrates with different stiffnesses is linked to its actin-regulatory activity, and possibly to its role in early onset osteoporosis, we made use of three PLS3 point mutants that were identified in patients and that were recently shown to display defects in their calcium-sensitive actin-bundling activity, namely, PLS3 A368D, PLS3 N446S and PLS3 L478P (Schwebach et al., 2020). The same study also showed that overexpression of PLS3 A368D increases FA size and density (number of FAs/100/μm^2^), whereas PLS3 N446S showed similar FA lengths and numbers as WT PLS3 (Schwebach et al., 2020). However, this experiment was done in U2OS osteosarcoma cells that were plated on glass and that were expressing endogenous levels of PLS3, raising the question how these mutants would behave in the absence of WT PLS3 and in cells plated on substrates with different stiffnesses. As such, we first investigated whether expression of EGFP-tagged versions of WT PLS3 or the osteopathogenic PLS3 mutants alter FA size or FA organization in a PLS3 KD background in cells plated on glass. As reported before, PLS3 L478P showed a diffuse cytoplasmic localization, whereas PLS3 A368D almost exclusively localized to FAs (Figure 4A). PLS3 N446S showed a localization pattern similar to WT PLS3 and was detected in the leading edge as well as in FAs (Figure 4A). Despite these differences in cellular localization, immunostaining with paxillin next showed that expression of the osteopathogenic PLS3 mutants did not change FA length of PLS3 KD cells (Supplementary Figure S4). A small non-significant increase in FA length was measured in PLS3 KD cells expressing WT PLS3 compared to empty EGFP (Supplementary Figure S4). These results are in line with our previous observations that PLS3 only has modest effects on FAs in MC3T3 cells plated on glass (Figures 2E, F). Interestingly, line scan analysis of the mutants that localize to FAs did show that PLS3 A368D overlaps strongly with actin but less with paxillin compared to WT PLS3 and PLS3 N446S (Figure 4B). Comparison of the distance to reach maximal PLS3 intensity in FAs confirmed that PLS3 A368D peaks around 1.3 μm, whereas WT PLS3 and PLS3 N446S peaked between 0.78–0.85 μm (Figure 4C). In addition, maximal paxillin and actin signals were reached slightly earlier and later, respectively, in FAs in cells expressing PLS3 A368D than in cells expressing WT PLS3 and PLS3 N446S. As a result, the distance between the paxillin and actin peak increased substantially in the presence of PLS3 A368D (0.85 μm) compared to WT PLS3 or N446S PLS3 (0.38–0.51 μm). Given that we did not observe any differences in FA length, this suggests that PLS3 does play a role in mechanotransduction but rather acts downstream from FAs, perhaps by regulating actomyosin-driven contraction of stress fibers and/or by activating downstream signaling pathways.

**FIGURE 5:**
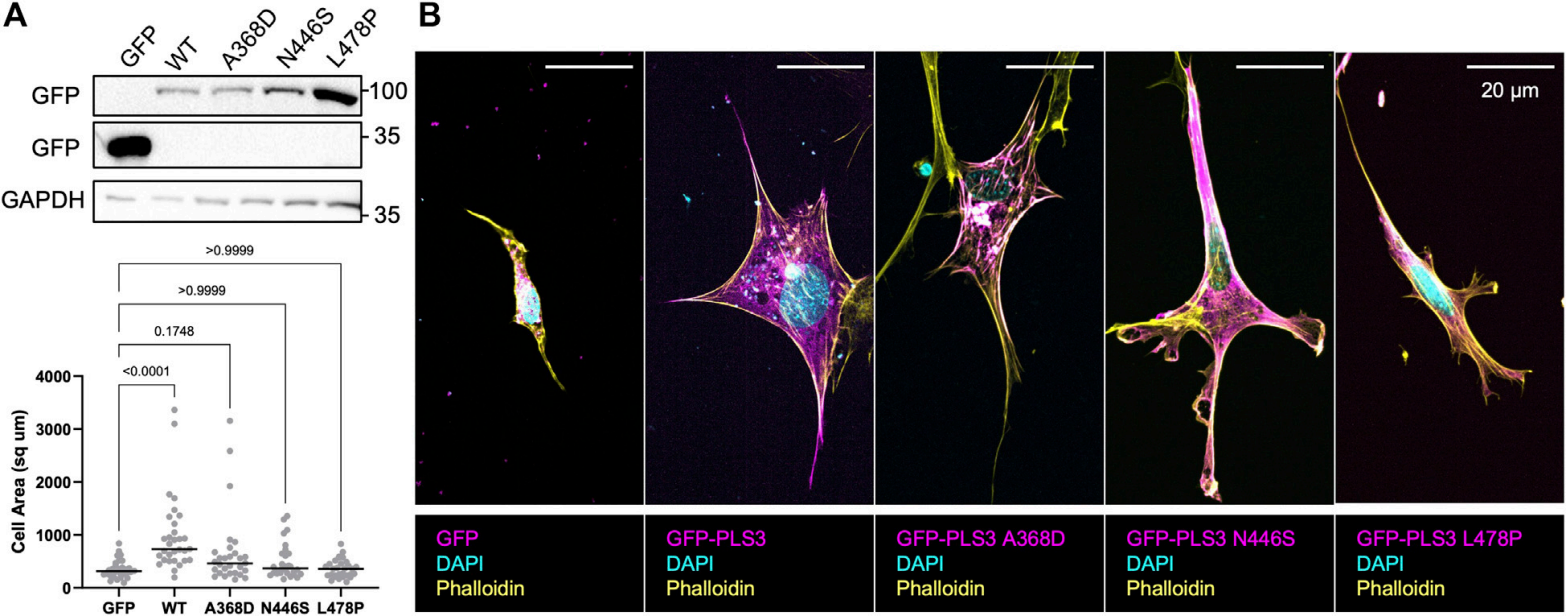
Osteopathogenic PLS3 mutants cannot rescue defects in cell size**. (A)** Western blot against GFP showing transient expression of empty GFP, GFP-tagged WT PLS3 and GFP-tagged osteogenic PLS3 mutants in PLS3 KD cells. GAPDH was used a loading control. **(B)** Representative confocal images of PLS3 KD cells expressing the indicated GFP construct plated on 100 kPa polyacrylamide hydrogels immunostained with DAPI (cyan) and phalloidin (yellow). **(C)** Distribution of cell area of PLS3 KD cells plated on 100 kPa polyacrylamide hydrogels expressing the indicated GFP construct. Cells were analyzed from three independent experiments with ≥9 cells per experiment. Statistical significance was determined using one-way ANOVA on ranks and Kruskal Wallis multiple comparison tests.

However, our previous observations also showed that PLS3 KD cells specifically behaved different when plated on substrates with a stiffness corresponding to unmineralized bone (100 kPa), resulting in significant changes in cell size. Thus, to further characterize how PLS3 contributes to osteoblast mechanosensation, we next transiently expressed EGFP-tagged WT PLS3 or the osteogenic PLS3 mutants in PLS3 KD cells plated on substrates with a stiffness corresponding to unmineralized bone. Analysis of cell size next showed that EGFP-PLS3 significantly increases cell size in comparison to PLS3 KD cells that express a control EGFP plasmid (Figures 5B, C). By contrast to WT PLS3, none of the osteopathogenic PLS3 mutants increased cell size of PLS3 KD cells plated on 100 kPa hydrogels (Figures 5B, C). Western blotting and staining for GFP showed similar expression levels of each construct, excluding that the lack of change in cell size was due to lower expression of the osteopathogenic PLS3 mutants (Figure 5A). This result does not only suggest that PLS3 regulates the response of osteoblasts to substrate stiffness, but also demonstrates that this process relies on the calcium-sensitive actin bundling activity of PLS3. Our observation that the osteopathogenic PLS3 mutants failed to rescue osteoblast spreading and adhesion, which is an important factor driving late osteoblast mineralization, further begins to elucidate how PLS3 contributes to bone health and by extension, to bone defects such as osteoporosis.

## Discussion

To date, a strong correlation has been established between early onset osteoporosis and mutation of PLS3, however the underlying mechanism, as well as how PLS3 contributes to general bone health and maintenance, is not understood. Given its predominant expression in mesenchymal lineage cells, including osteoblasts and osteocytes, this study focused on characterizing the role of PLS3 in osteoblasts. For this purpose, we developed a stable PLS3 KD cell line using MC3T3-E1 pre-osteoblasts. Histological analysis showed that depletion of PLS3 specifically affects deposition of hydroxyapatite mineral (Figure 1), suggesting that PLS3 plays a role in the later stages of osteoblast mineralization. This is in line with the delayed mineralization phenotype observed in biopsy samples from patients with PLS3 mutations and the decreased mineral deposition detected in cortical osteoblasts isolated from PLS3 knockout mice (Laine et al., 2015; Kämpe et al., 2017a; Yorgan et al., 2020). It further emphasizes that the pathophysiology underlying PLS3-related osteoporosis differs from other forms of congenital osteoporosis arising from impaired collagen production and/or transport (Mäkitie and Zillikens, 2022). This warrants a better understanding of the mechanism underlying osteoporosis linked to PLS3 mutations and calls for novel *in vitro* model systems such as we have established in this study.

Our results further show that PLS3 KD cells respond differently to substrates with a stiffness that corresponds to unmineralized bone or osteoid (∼100 kPa) compared to control cells, whereas we only observed small non-significant changes in their response when plated on glass (∼ GPa or fully mineralized bone) or softer substrates that mimic endothelial tissue (6 kPa) (Figures 2, 3). We hypothesize that these differences are in part related to the cell type we used and the fact that we used Poly-L instead of a collagen coating for our experiments on glass. Under these conditions, MC3T3 cells were shown to form fewer FAs and stress fibers than when plated on glass coverslips coated with collagen (Takai et al., 2005), indicating that different downstream signaling pathways are activated. This could explain why we observed similar but non-significant trends in cell size and FA length in cells plated on glass (plain or coated with diluted Matrigel) *versus* cells plated on Matrigel-coated hydrogels. Independent of experimental set-up, our data further suggests that PLS3 plays a role at a very specific stage during mineralization. In line with this, Fahiminiya and colleagues showed that both PLS3 mRNA and protein expression doubled between day 10–16 of MC3T3 differentiation, after which PLS3 mRNA concentration decreased whereas PLS3 protein concentration remained similar (Fahiminiya et al., 2014). Alizarin red stain demonstrated that the changes in the protein level of PLS3 coincided with increasing mineralization. Taken together with our results, it would be of future interest to test how the expression of PLS3 changes in osteoblasts as well as in other cell types plated on substrates with increasing stiffness or a different ECM composition (e.g., collagen I *versus* fibronectin, etc.). This would not only bring insight into potential pathways and extracellular cues that regulate PLS3, but also render insight into how specific this PLS3 response is for osteoblast cells, which would further shed light onto how mutation of a ubiquitously expressed protein such as PLS3 specifically induces a bone defect. In addition, PLS3 is also expressed by osteocytes (Wesseling-Perry et al., 2017), which sense mechanical loading through fluid shear stress (FSS) rather than substrate stiffness (Capulli et al., 2014; Uda et al., 2017). This raises the question whether PLS3 plays a role in regulating the response to different mechanical stimuli. In line with this, PLS3 was shown to accumulate in osteocyte processes after exposure to FSS *in vitro* as well as to be downregulated in knockout mice of the mechanosensitive calcium channel, Piezo1 (Kamioka et al., 2004; Li et al., 2014). Altogether, these are good arguments to investigate the role of PLS3 in osteocyte mechanosensation, especially in the light that PLS3 knockout mice did not show any defects in osteocyte number or architecture (Neugebauer et al., 2018; Yorgan et al., 2020). As osteocytes regulate both osteoblast as well as osteoclast activity, a role of PLS3 in osteocytes could also explain the osteoclast defect reported in PLS3 knockout mice (Neugebauer et al., 2018).

In line with a decrease in cell size, our data further demonstrates that PLS3 KD cells were smaller in size and displayed a smaller number of FAs, suggesting that the mechanosensitive response of PLS3 KD cells to increasing mineralization of the ECM might be disrupted. In line with our observations, depletion of PLS3 in the mouse epidermis affected epidermal organization but not differentiation (Dor-On et al., 2017). The authors further showed that this phenotype was not due to altered expression of genes involved in adhesion (β1-integrin, ILK, β-actin, Myosin IIA, and E-Cadherin) or basal membrane composition (laminin, collagen), but to defective regulation of actomyosin contraction. Based on their results and earlier work that showed that plastin in lower eukaryotes stimulates actomyosin contractility, they postulated that PLS3 affects epidermal architecture by regulating keratinocyte mechanotransduction (Reichl et al., 2008; Ding et al., 2017; Dor-On et al., 2017). Even though osteoblast mineralization and skin development are very different processes, this does raise the question whether the decreased mineralization phenotype in PLS3 KD osteoblasts is secondary to decreased actomyosin-mediated contraction, which could lead to defective activation of downstream signaling pathways that directly or indirectly regulate mineralization (Clark et al., 2007; Zaidel-Bar et al., 2015; Hall et al., 2019; Khan et al., 2020). Alternatively, it was recently shown that osteoblast themselves can rearrange the collagen matrix, presumably to shape the osteocyte lacunae (Lu et al., 2018; Shiflett et al., 2019). However, it cannot be excluded that this osteoblast-mediated remodeling contributes to the hierarchical organization of collagen that is required to obtain optimally mineralized bone (Salvatore et al., 2021; Ma et al., 2022). Rearrangement of the ECM is tightly connected to actomyosin contractility and FA spacing, suggesting that PLS3 KD osteoblasts might be able to deposit a collagen matrix, but fail to properly organize it, thereby resulting in its inadequate mineralization. In line with this, PLS3 was recently shown to be indispensable to bridge gaps in a discontinuous fibronectin matrix (Garbett et al., 2020). It is conceivable to hypothesize that PLS3 similarly helps cells cross gaps in a collagen matrix, again thus contributing to better cell spreading and mineralization of the underlying matrix. As such, analysis of our PLS3 KD cells at different stages during mineralization using a combination of advanced imaging techniques, including traction force microscopy to monitor actomyosin contraction and electron microscopy and polarized light microscopy to visualize collagen organization, should further clarify the mechanism underlying the role of PLS3 in osteoblast mineralization and will be the focus of future work.

Finally, we show that three ostepathogenic PLS3 mutants with aberrant actin-bundling activity, fail to rescue the cell size defect in PLS3 KD cells plated on substrates with a stiffness of unmineralized bone (Figure 5). On the one hand, this indicates that actin-bundling by PLS3 plays a role in (osteoblast) mechanosensation. However, how this is regulated by calcium remains to be elucidated. Previous work showed that F-actin bundling by PLS3 A368D and PLS3 N446S *in vitro* is hypo- and hypersensitive to calcium, respectively (Schwebach et al., 2020). In cells, the same study demonstrated that calcium depletion increased the lifetime of WT PLS3 and PLS3 N446S localized to FAs, whereas the lifetime of PLS3 A368D was unaltered (Schwebach et al., 2020). This suggests that calcium binding not only reduces F-actin bundling by PLS3, but might also induce PLS3 dissociation from FAs. How this relates to the role of PLS3 in mechanosensation as well as in promoting osteoblast mineralization remains to be investigated. However, given that calcium also directly regulates the turnover of FAs, a more thorough study is needed to establish whether these observations are due to an effect of calcium on PLS3 directly or on other FA proteins. On the other hand, our data also begins to shed light onto how PLS3 mutation leads to defects in osteoblast mineralization, which potentially could explain how PLS3 promotes general bone health and maintenance, but also might play a role under pathophysiological conditions such as during bone repair or the development of osteoporosis. Our PLS3 KD cells in combination with hydrogels with stiffnesses between 6–100 kPa provide an important tool and technical framework to further probe the role of PLS3 in these processes.

## Materials and methods

### MC3T3-E1 cell culture

Murine calvarial osteoblast cells MC3T3-E1 subclone 4 were purchased from ATCC and were cultured at 37°C in a humidified atmosphere with 5% CO2 in growth media consisting of MEMα supplemented with 10% FBS and penicillin-streptomycin (Thermo Fisher Scientific, Waltham, MA). For overexpression experiments, 300,000 cells were electroporated with 2 μg of each of the indicated plasmids and subsequently plated on either Poly-D lysine treated 3.5 cm imaging dishes or on hydrogels (Lonza Biosciences, Basel, Switzerland).

### Stable MC3T3-E1 cell lines

Scrambled and PLS3 knockdown cell lines were generated using a lentiviral shRNA approach described by Dor-On et al. (2017). Briefly, shRNA sequences were cloned into pLKO.1 neo (addgene plasmid #13425, Gift from Sheila Stewart) using AgeI and EcoRI restriction sites. The following shRNAs were used: 5′-ccg​gtG​CTC​AGA​ATT​TAG​ACG​GGA​ATc​tcg​agA​TTC​CCG​TCT​AAA​TTC​TGA​GCt​ttt​tg-3′ (MmPLS3), and 5′-ccg​gtG​CAG​TTA​AGA​GTG​AGA​CTA​CTc​tcg​agA​GTA​GTC​TCA​CTC​TTA​ACT​GCt​ttt​tg-3’ (non-targeting or scrambled shRNA) adapted from (Dor-On et al., 2017). To produce lentivirus, HEK293T cells were grown to 90% confluency in growth media consisting of DMEM glutamax high glucose supplemented with 10% FBS and transfected with third generation lentivirus packaging plasmids (pMDL/pRRE, pMD2. G, and pRS-REV) and pLKO.1 PLS3 or scrambled shRNA plasmids using TransIT-LT1 transfection reagent (MirusBio, Madison, WI). Next day, media was replaced with growth media supplemented with 30% FBS. Three days after transfection, the medium was filtered through a 0.45 µm filter and lentivirus was harvested via centrifugation for 2 h at 16,000 x g. The resulting lentiviral pellet was resuspended in PBS and stored in aliquots at −80°C. To generate stable cell lines, lentivirus was added directly to MC3T3 cells at 60% confluency and were subsequently selected at 0.5 mg/mL of G418 for 2 weeks. PLS3 knockdown was confirmed using Western blotting (Figure 1B).

### Molecular cloning

EGFP-PLS3 was cloned by introducing the PLS3 gene using EcoRI and BamHI restriction sites and confirmed by sequencing. Osteogenic PLS3 mutants (PLS3 A368D, PLS3 N446S and L478P) were generated using site-directed mutagenesis (Agilent, Santa Clara, CA) and were confirmed by sequencing. EGFP-PLS3 was cloned into the sites of pBOB-GFP (Addgene plasmid #12337) and EGFP-PLS3 to produce lentivirus for rescue experiments.

### Differentiation assays

For differentiation assays, cells were plated at 2,000 cells per square centimeter and allowed to grow to confluence prior to differentiation. To stimulate differentiation, cells were grown in growth media supplemented with 100 uM ascorbic acid and 2 mM β-glycerophosphate, changing media every 3 days. After 1 week of differentiation, alkaline phosphatase staining and enzymatic activity were determined. For staining, cells were fixed in 4% PFA in PBS for 10 min, washed extensively with PBS, then with TRIS-buffered saline (TBS) pH 8, before staining with 1 step NBT/BCIP solution for 30 min at 37°C (ThermoFisher, Saint Louis, MO). Cells were subsequently washed with deionized water, air dried, and imaged. For enzymatic assays, Sensolyte PnPP alkaline phosphatase colorimetric assay kits (AnaSpec, Fremont, CA) were used according to manufacturer’s instructions. Briefly, cell monolayers were washed with ice cold assay buffer, scraped into microfuge tubes, and lysed using assay buffer supplemented with 0.1% triton. Resulting lysates were precleared at 20,000 g for 20 min at 4°C. Bradford assays were used to measure protein concentrations and 8 ug of lysate was added to 100 μL reactions containing 50 μL of pNPP substrate. For the standard curve, calf intestinal phosphatase (CIP, New England BioLabs, Ipswich, MA) was added at the following amounts to 100 μL reactions: 0, 0.15, 0.3, 0.6, 1.2, 2.5, 5, and 10 ng. Samples were allowed to incubate for 1 h at room temperature. A plate reader was used to measure absorbance at 405 nm and alkaline phosphatase activity was interpolated to the CIP standard curve. After 2 weeks of differentiation, cells were harvested for picrosirius red and alizarin red staining. To measure collagen deposition, cells were washed with PBS, fixed with Bouin’s fixative (Electron Microscopy Sciences, Hatfield, PA) for 1 h, washed with PBS, and allowed to dry. Cells were subsequently stained with 0.1% sirius red in picric acid for 1 hour, rinsed with 0.5 M acetic acid, washed extensively with deionized water, and allowed to dry overnight. Representative images were captured using a flatbed scanner, then bound dye was eluted with 0.5 M sodium hydroxide. A plate reader was used to measure absorbance at 590 nm and interpolated against a rat collagen standard curve (0, 10, 25, 50, and 100 ug). To measure mineral deposition, cells were washed with PBS, fixed with ice cold 70% ethanol for 1 h, allowed to dry. Cells were subsequently stained with 40 mM alizarin red pH 4.1 for 30 min, washed with deionized water, and allowed to dry overnight. Representative images were captured using a flatbed scanner, then bound dye with eluted with 10% glacial acetic acid. A plate reader was used to measure absorbance at 405 nm and interpolated against an alizarin standard curve (0, 0.1, 0.25, 0.5, 1, 2 mM).

### Hydrogels

To generate polyacrylamide hydrogels of different stiffness, plasma-cleaned coverslips were coated with 0.4% silane (Sigma, Burlington, MA) in milliQ water (pH 3.5) for 1 hour. Ice cold basal media was used to dilute thawed Matrigel (Corning, Saint Louis, MO) Plasma-cleaned coverslips were coated with diluted Matrigel for 1 h, after which excess silane and Matrigel were removed. Polyacrylamide hydrogel solutions of the indicated stiffnesses were prepared according to published protocols (Engler et al., 2006; Tse and Engler, 2010). For assembly, 15 μL of freshly prepared hydrogel solution was added to a silanized coverslip and then promptly covered with a Matrigel coated coverslip. Following polymerization of the hydrogel, assembled hydrogels were placed in PBS for at least 1 h prior to cell seeding. To plate cells on the hydrogels, the coverslip that was originally coated in Matrigel was removed from the gel using a razor blade and submerged with the Matrigel-coated side face-up in standard growth medium.

**Table:** 

Hydrogel mix (μL)	6 kPa	10 kPa	35 kPa	100 kPa
milliQ water	714	689	589	14
Acrylamide (40%)	250	250	250	375
Bis-acrylamide (2%)	25	50	150	600
TEMED	1	1	1	1
1% APS	10	10	10	10

### Immunofluorescence

For immunofluorescence assays, 15,000 cells were seeded on glass coverslips, Matrigel-coated glass coverslips, or Matrigel-coated hydrogels. Twenty-4 hours after seeding, cells were fixed with 4% PFA in PBS for 10 min, washed extensively with PBS, permeabilized with 0.1% Triton X-100 in PBS for 10 min, washed extensively with PBS, and blocked in 2.5% BSA in PBS for 1 h. Cells were incubated overnight at 4°C with the indicated primary antibody diluted 1:200 in blocking buffer. The following primary antibodies were used for immunofluorescence staining: Paxillin (BD Biosciences, Franklin Lakes, NJ), vinculin (Novus Biologicals, Littleton, CO), PLS3 (ThermoFisher, Saint Louis, MO). Next day, cells were washed in PBS and incubated with the secondary antibody (ThermoFisher, Saint Louis, MO) at 1:1,000, phalloidin (ThermoFisher, Saint Louis, MO) at 1:400, and DAPI at 1:10,000 in blocking buffer and washed extensively in PBS. Hydrogels and coverslips were mounted with anti-fade (ThermoFisher, Saint Louis, MO).

### Western blotting

Cells for Western blotting were washed with PBS and harvested in lysis buffer containing 20 mM TRIS pH 7.4, 0–150 mM NaCl, 1% triton, 1x EDTA-free protease inhibitors, 1 mM PMSF, and benzamidine. For differentiated cells, 0 mM NaCl was used and for non-differentiated cells, 150 mM NaCl was used. Cells were allowed to lyse over ice for 30 min, flash-frozen in liquid nitrogen, thawed on ice, then precleared for 15 min at 20,000 g at 4°C. Protein concentrations were determined using bradford assays and 20 ug were loaded onto SDS-PAGE gels. Samples were transferred onto PVDF membranes (Millipore Sigma, Burlington, MA) activated in 100% methanol in 1x transfer buffer containing 15% methanol at 100 V for 1 h at 4°C. Blots were allowed to dry, blocked for 1 h in TBS supplemented with 5% BSA and 0.1% Tween. Blots were incubated with specified antibodies (see immunofluorescence section) using GAPDH as an equal loading control (Santa Cruz Biotechnologies, Santa Cruz, CA) overnight at 4°C. Blots were washed extensively with TBS-T (0.1% tween), incubated with horseradish peroxidase-linked anti-mouse and anti-rabbit immunoglobulin G secondary antibodies (Cell Signaling Technology, Danvers, MA) for 1 h at room temperature. Blots were washed extensively and developed using clarity western ECL substrate (Bio-Rad Laboratories, Hercules, CA) and imaged using a Gel-Doc Imager (Bio-Rad Laboratories, Hercules, CA).

**Table:** 

Antibody	Company and catalog #
Plastin-3	Invitrogen, PA5 27,883
GAPDH	Santa Cruz, 365,062
GFP	Santa Cruz, 9,996
Vinculin	Cell Signaling Technology, 139,015
Paxillin	BD Biosciences, 612,405

### Confocal imaging

All cells were imaged using a Nikon Ti2 inverted microscope equipped with a ×60 Plan-Apo oil immersion objective and a Yokogawa CSU-W1 spinning disk confocal attached to a Hamamatsu ORCA-FLASH4.0 CMOS camera. Cells were imaged in DMEM FluoroBrite (ThermoFisher Scientific) medium supplemented with 5% FBS at 37°C and 5% CO2. Images stacks were captured at 16-bit 2048 × 2044 resolution with an axial spacing of 0.2 mm using the Nikon Elements Software package. Cells were chosen at random for capture and the resulting files were blinded during image analysis. FIJI was used for image processing (https://imagej.net/Fiji). To generate focal adhesion line scans, the paxillin channel was used to create ROIs that measured twice the length of the given focal adhesion. Cell areas were determined using the phalloidin channel and multi-point polygon tool. An adapted methodology was used to quantify focal adhesion sizes as previously described (Horzum et al., 2014). Briefly, stacks were converted to maximal projections, background was subtracted with the rolling ball radius set to 25, local contrast was enhanced with CLAHE (Contrast Limited Adaptive Histogram Equalization, block size = 19, histogram bins = 256, maximum slope = 6, no mask, and slow), background was further reduced using mathematical exponential, and lastly brightness and contrast were adjusted automatically. Next, Log3D (Laplacian of Gaussian or Mexican Hat, sigma x = 5, sigma y = 5) filter was applied (bigwww.epfl.ch/sage/soft/LoG3D), threshold (triangle) command was applied to create a binary image, and the Analyze particles tool (size = 25 to infinity) was used to generate ROIs. The ROIs were assessed manually to visually confirm detection of focal adhesions. This methodology was compared against a manually acquired data set to confirm accuracy. To determine Pearson’s correlation coefficient, Coloc 2 tool in FIJI was performed using the indicated channels for each FA linescan using a 6 pixel wide area.

## Data Availability

The raw data supporting the conclusion of this article will be made available by the authors, without undue reservation.
