# Supplementary figures and images for: The actin-bundling protein, PLS3, is part of the mechanoresponsive machinery that regulates osteoblast mineralization
Source: Front Cell Dev Biol. 2023 Nov 27;11:1141738. doi: 10.3389/fcell.2023.1141738 (PMC10711096; doi:10.3389/fcell.2023.1141738)

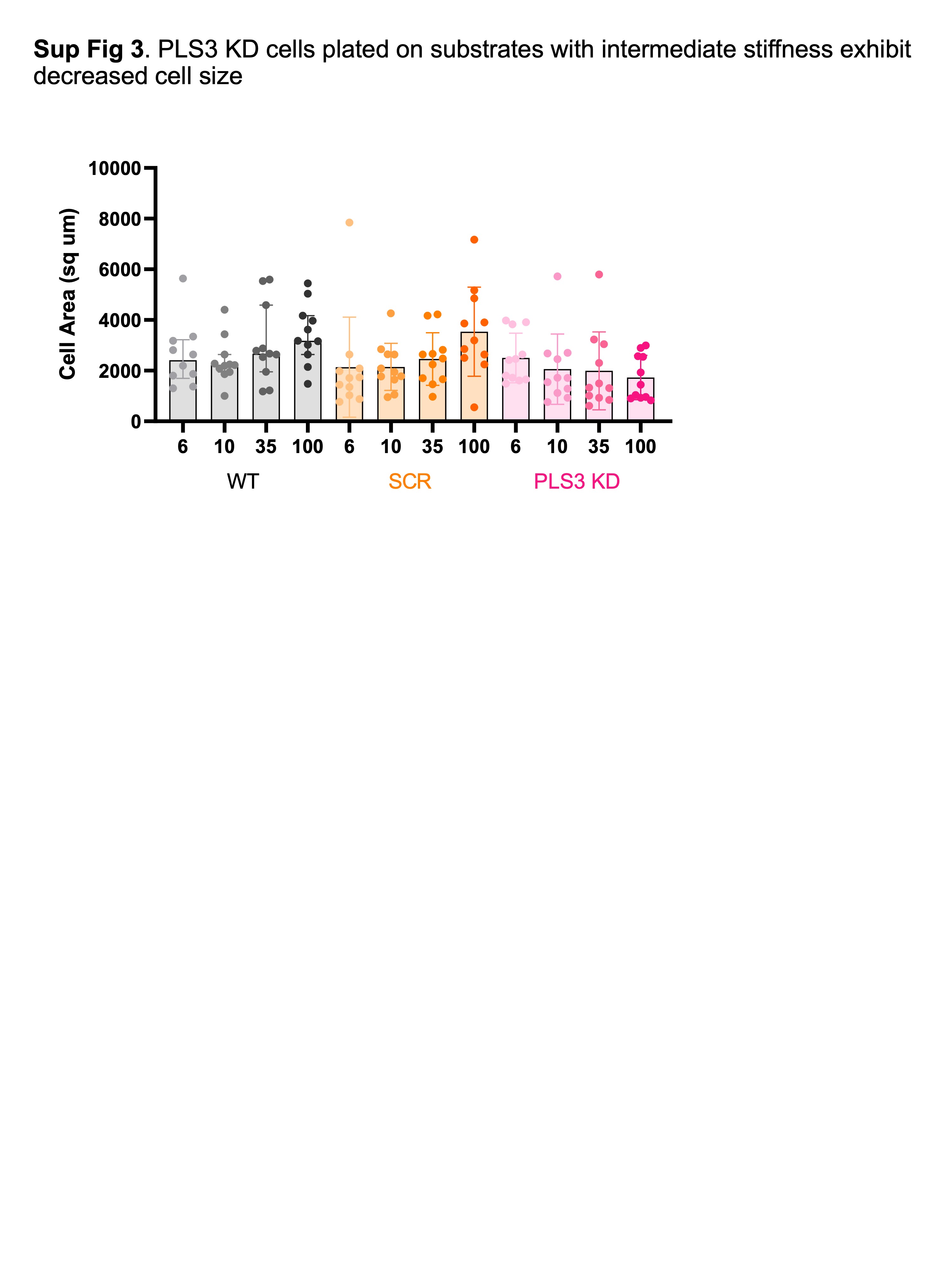

Supplement: Supplementary file 1 [file Image3.jpeg]

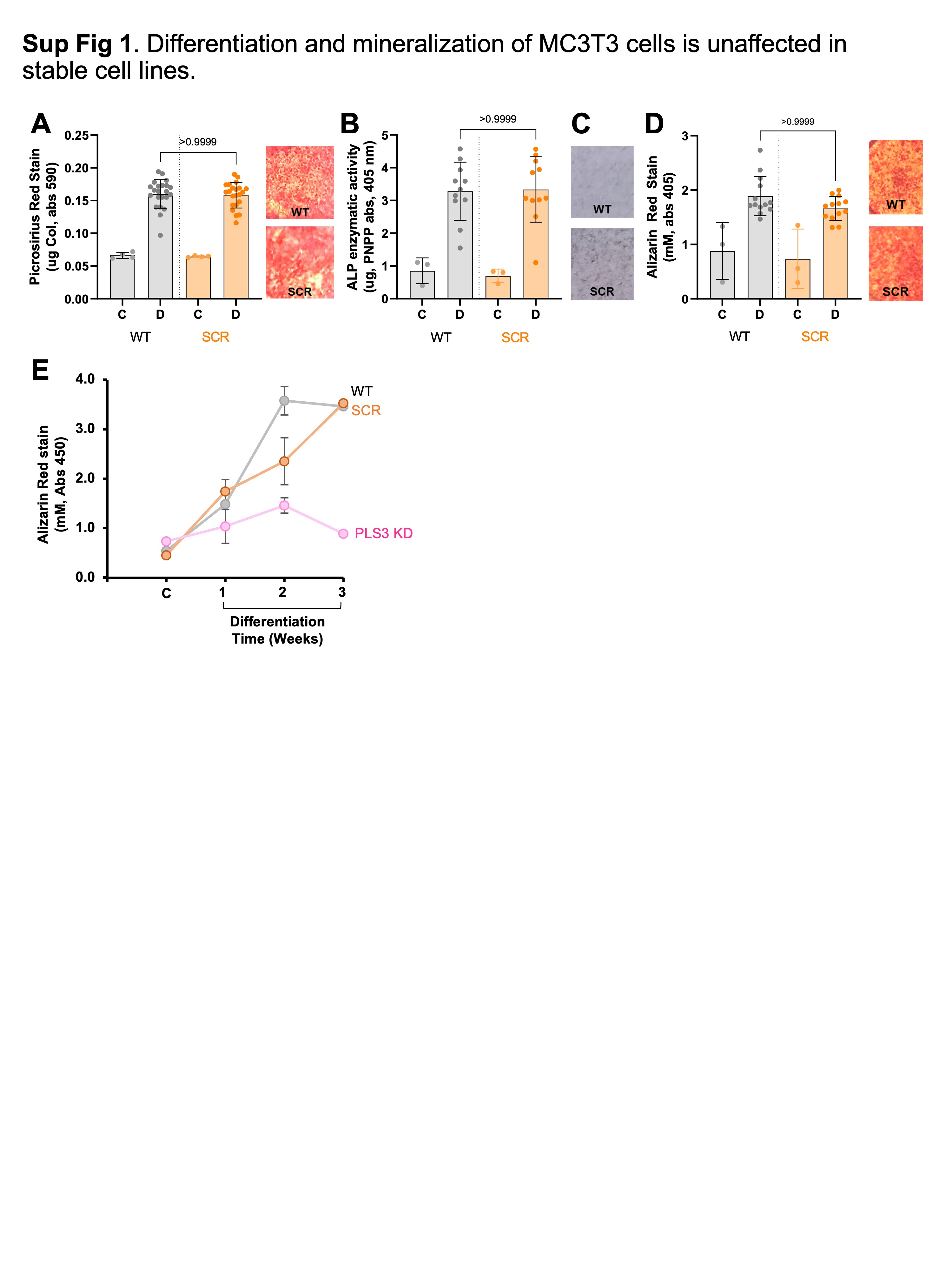

Supplement: Supplementary file 2 [file Image1.jpeg]

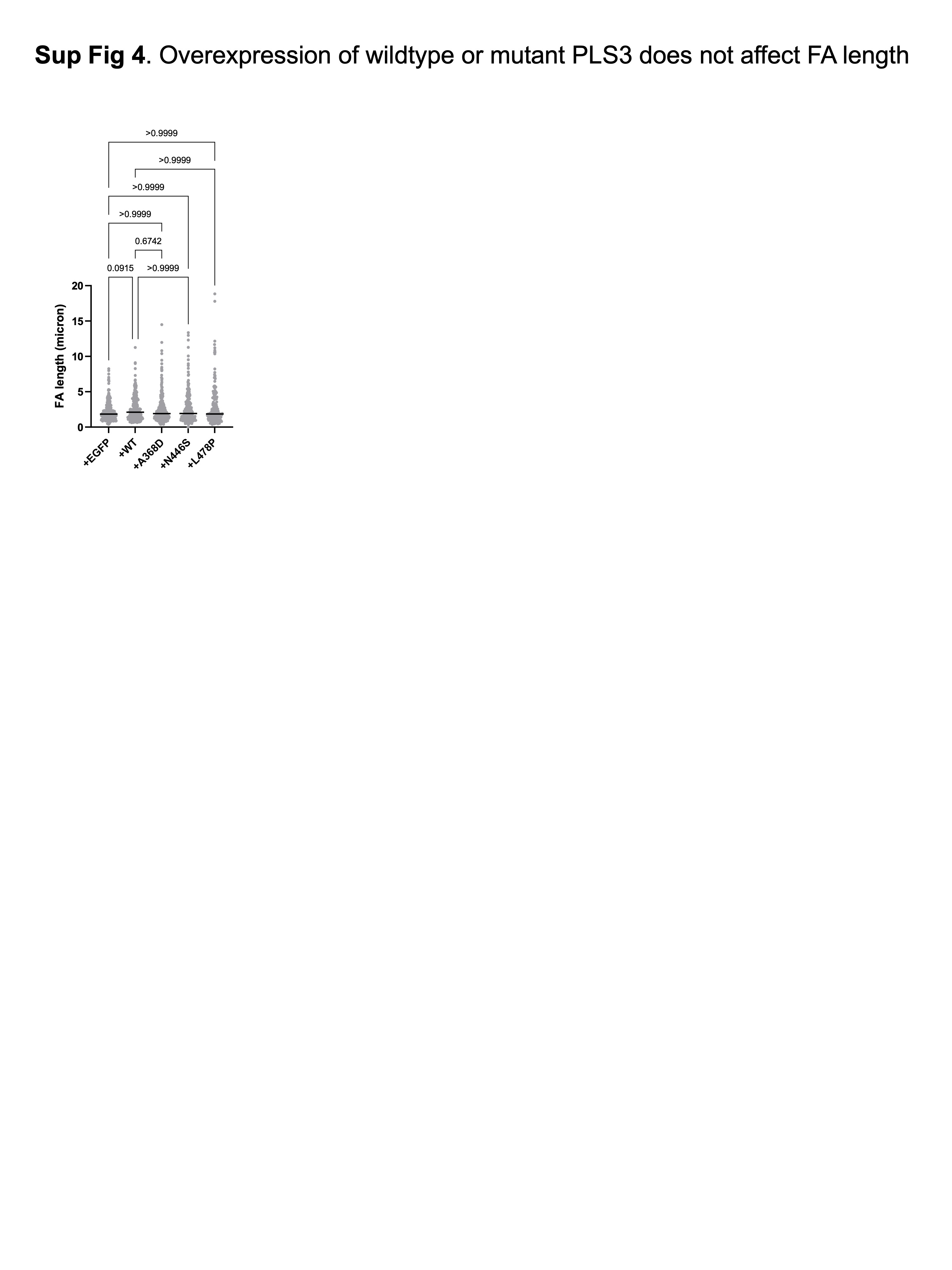

Supplement: Supplementary file 3 [file Image4.jpeg]

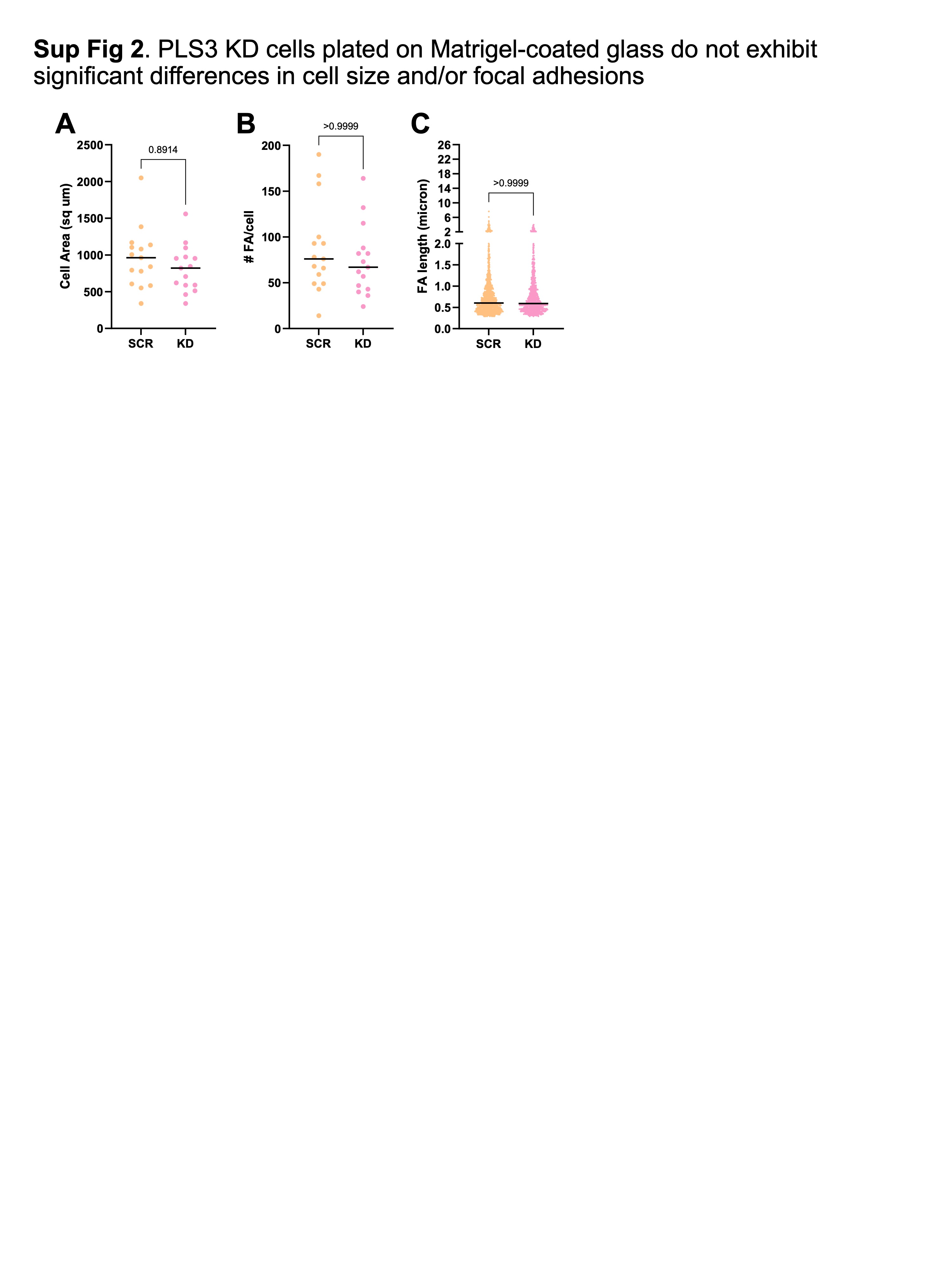

Supplement: Supplementary file 4 [file Image2.jpeg]
